# Chemically modified cysteine in CHO fed-batch processes and impact on the specific productivity

**DOI:** 10.1186/1753-6561-9-S9-P55

**Published:** 2015-12-14

**Authors:** Caroline Hecklau, Ronja Seibel, Maria Wehsling, Jennifer Kuschelewski, Aline Zimmer

**Affiliations:** 1 Merck KGaA, Frankfurter Straße 250, 64293 Darmstadt, Germany

## Background

Mammalian fed-batch processes for monoclonal antibody (mAb) production rely on strategic feeding of several nutrients such as glucose, vitamins and amino acids to extend culture time and improve protein production[1].In actual processes, L-cysteine and L-tyrosine are fed separately at alkaline pH due their low stability and low solubility at neutral pH, resulting in pH peaks and precipitations[2]. To simplify next generation processes, both amino acids have been chemically modified to enhance their respective stability and solubility profiles. Previous work has demonstrated that phosphotyrosine disodium salt (PTyr2Na) is a stable L-tyrosine derivative and can be used in neutral pH feeds without having a detectable impact on the culture performance or the mAb quality attributes[3]. Here, we present results obtained using a L-cysteine derivative in a neutral pH, single-feed system.

## Materials and methods

The stability of the L-cysteine derivative was evaluated in neutral pH, CellventoTM Feed-220 during three months at room temperature or 4°C.For fed-batch cultures, a CHO K1 clone expressing a human mAb was used. Cultivation was performed in the Cellvento™ CHO-220 media system according to the product process guidance. For controls, Cellvento™ Feed-220 and a separate alkaline cysteine/tyrosine feed were used whereas in the single feed process, the L-cysteine derivative and PTyr2Na were directly solubilized in the neutral pH, main feed. Experiments were performed in spin tubes and 1.2L bioreactors. Growth and viability were monitored using a ViCell®,titer was determined using Cedex Bio HT and specific productivity was calculated based on titer, integral viable cell density and dilution. For spent media analysis, the L-cysteine derivative and amino acid quantification was performed by UPLCusing AccQ·TagTMUltra Reagent. To assess the reactive potential of the feed, H2DCFDA was added to the neutral pH, Cellvento™ Feed-220 supplemented or not with the derivative. For intracellular reactive species quantification, cells were labeled with carboxy-H2DCFDA and fluorescence was measured. To evaluate the capacity of the cells to metabolize the derivative, cell lysates were spiked and formed products were quantified by UPLC. For mAb analysis, N-glycosylation was quantified using HPLC after 2-AB labeling, whereas charge variants were determined using cIEF.

## Results

Analysis of the L-cysteine derivative stability in neutral pH feed indicated no change in the derivative concentration nor L-cysteine/L-cystine release when stored over three months at room temperature or 4°C. No precipitation or color change was observed indicating that the derivative was stable under the tested conditions. Fed-batch cultivation in spin tubes with the single-feed strategy resulted in higher final viabilities leading to enhanced final titers when compared to the control condition. Spin tube results were confirmed in bioreactors leading to higher final viabilities, increased titers and higher specific productivity with the single feed strategy. Lower dye oxidation was measured after addition of H2DCFDA to neutral pH, Cellvento™ Feed-220 containing the derivative compared to feed alone indicating lower reactive potential. Lower intracellular dye oxidation was detected by addition of carboxy-H2DCFDA to spin tube single-feed fed-batch cultures indicating lower intracellular reactive species generation when using the derivative. When spiked into cell lysates, the cysteine derivative was metabolized to cysteine. No difference in N-glycosylation or charge variant was detected in the produced mAb indicating no influence of the derivative on the presented critical quality attributes.

## Conclusions

This study demonstrates that the L-cysteine derivative and PTyr2Na can be integrated into a neutral pH feed and can be used as a source of L-cysteine in fed-batch processes leading to a higher specific productivity compared to the state-of-the-art process without affecting mAb critical quality attributes.
